# Facial Recognition in a Group-Living Cichlid Fish

**DOI:** 10.1371/journal.pone.0142552

**Published:** 2015-11-25

**Authors:** Masanori Kohda, Lyndon Alexander Jordan, Takashi Hotta, Naoya Kosaka, Kenji Karino, Hirokazu Tanaka, Masami Taniyama, Tomohiro Takeyama

**Affiliations:** 1 Laboratory of Animal Sociology, Department of Biology and Geosciences, Graduate School of Sciences, Osaka City University, Sumiyoshi, Osaka 558–8585, Japan; 2 Department of Biology, Tokyo Gakugei University, Koganei, Tokyo 184–8501, Japan; University of Natural Resources and Life Sciences, Vienna, AUSTRIA

## Abstract

The theoretical underpinnings of the mechanisms of sociality, e.g. territoriality, hierarchy, and reciprocity, are based on assumptions of individual recognition. While behavioural evidence suggests individual recognition is widespread, the cues that animals use to recognise individuals are established in only a handful of systems. Here, we use digital models to demonstrate that facial features are the visual cue used for individual recognition in the social fish *Neolamprologus pulcher*. Focal fish were exposed to digital images showing four different combinations of familiar and unfamiliar face and body colorations. Focal fish attended to digital models with unfamiliar faces longer and from a further distance to the model than to models with familiar faces. These results strongly suggest that fish can distinguish individuals accurately using facial colour patterns. Our observations also suggest that fish are able to rapidly (≤ 0.5 sec) discriminate between familiar and unfamiliar individuals, a speed of recognition comparable to primates including humans.

## Introduction

Lake Tanganyika cichlids show a great diversity of colour patterns among populations and species, and the divergence in visual signals can function in species recognition (e.g. [1, 2]). Stabilizing selection on signal traits in this case can arise through selection against hetero-specific mating [2], or through benefits of association with conspecifics (e.g. predator confusion effects, [3]). Signals can also serve to distinguish sexes within a species, being commonly a result of sexual selection [4]. This generally leads to sexually polymorphic traits, most often where males display conspicuous sexual traits that are used in courtship or competition for mates [5, 6, 7]. These forms of recognition typically lead to monomorphic traits at the species level, either in the visual, auditory, or chemical channels.

Even when signals are largely conserved, communication signals can be multimodal, and different functional elements may vary among individuals while others are consistent throughout the population. Thus, although an individual may have monomorphic species level traits (e.g. so called ‘poster colouration’) [1], variation in other traits may allow for individual recognition [8]. Individual recognition is implied in a broad range of studies, especially in social than solitary animals [8, 9], yet the traits responsible for this recognition have been identified in only a fraction of cases [8, 9, 10, 11]. When the same individuals interact repeatedly for a long period, recognition of individuals can play an important role in the avoidance of costs associated with agonistic interactions and the maintenance of stable social structure. Generally, individual recognition is more pronounced among highly social species [8, 9, 10, 11, 12, 13], but the actual traits responsible for this recognition is poorly understood. In particular, studies on the visual cues used by animals to distinguish between familiar and unfamiliar social group members are taxonomically restricted, having only been described for some mammals (e.g. [14, 15, 16]), birds [17], and remarkably for paper wasps [9, 10, 11, 18]. In these described cases, features of facial colouration or pattern are the primary signal, although other cues might contribute to individual identification.

**Fig 1:**
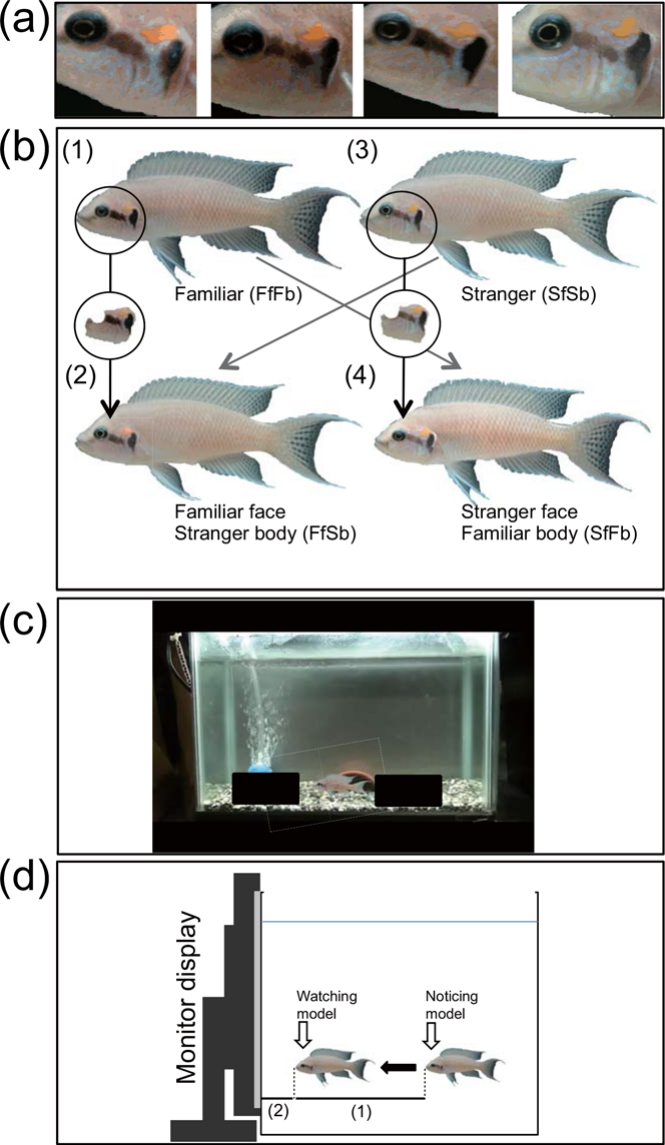
Facial colour patterns and four types of models of *Neolamprologus pulcher*, and their presentation in experiments. (a): facial colour pattern of four individual fish. (b): preparation of four types of digital-models; (FfFb): familiar neighbour, (FfSb): familiar neighbour’s face on stranger body, (SfSb): stranger and (SfFb): stranger’s face on familiar neighbour body. (c): monitor display exhibiting moving models in an experimental tank. (d): typical reaction of fish just after noticing an emerging model; (1) distance of fish approaching a site for watching model, and (2) distance between site of fish watching model and model on screen.

Recently, many examples of social cognitive abilities have been documented in fish, occasionally to a level of sophistication comparable to that of mammals and birds (for reviews [12, 13, 19; 20, 21]). For example, many social fish recognize individual group members visually (e.g. [13, 21]), but the visual signals they use is unknown. One of the leading experimental systems for the study of social behaviour is the African cichlid *Neolamprologus pulcher* [22, 23], a fish that lives in large family groups organized by dominance hierarchy (5–15 group members). *N*. *pulcher* can recognize group members using only visual cues [13, 24, 25], and distinguish familiar neighbours of the adjacent territory from strangers (i.e. dear enemy relationship) [26]. They inhabit open water benthic habitats near the lake shore with good water transparency (down to 30 m depth). The cues for individual recognition such as chemically mediated cues are unlikely used in social communication [27]. The visual signals they use for individual identification are unknown, but the variations in facial colour patterns among individuals of *N*. *pulcher* (Fig 1A and 1B) suggest a role for facial colour patterns in recognition. We hypothesize that this variability in facial colour pattern may play a role in individual recognition independently of other more conserved visual cues like body shape and overall colouration in this fish.

Fish may have visual acuity comparable to humans [28], and also respond to video playback in a manner comparable to live stimuli [29]. *N*. *pulcher* also respond to the video images of conspecifics, discriminating familiar fish and unfamiliar fish [25]. Here, we tested the role of facial recognition by generating familiar and unfamiliar pairs of individuals and using digital images modified to separate the effects of facial colouration and body characteristics on individual recognition behaviour.

## Materials and Methods

Study fish were obtained from commercial breeding stocks, and kept in 10 stock tanks at 26 C° under 12:12 h light-dark cycles in the laboratory of Osaka City University. Fish were fed with commercial flake food (Tetramin) twice a day. Sexually active males of similar size (42–49 mm in standard length) that were used for experiments were videotaped using Sony HDR-CX370 video cameras.

### Procedure of experiment 1

To create familiar neighbours, we followed Frostman & Sherman 2004 [26]: two size-matched males (≤ 3 mm in size difference) were taken from stock tanks that had been separated for over one year and placed into two laterally adjacent 30 x 17 x 23 (Height) cm^3^ aquaria (*n* = 8 males of four combinations: each male had its respective neighbour male) that allowed visual contact. These experimental tanks contained a half cut flowerpot and air-stone on a 3 cm thick substrate of small pebbles (Fig 1C). Males were size-matched, with differences in size consistent with preceding studies on this species [26]. Duration of aggression of males across the glass dividers was recorded for 20 min a day for seven days. An unfamiliar ‘stranger’ male was presented on the 10th day to each focal individual after the removal of the familiar neighbour male, and the behavioural responses were again recorded. In previous studies [26], focal males attacked males newly introduced in the adjacent tank but aggression decreased over the course of a week and was subsequently rarely observed. On introduction of a new and unfamiliar male, they once again became aggressive. This reduction of aggression against neighbouring males is known as the ‘dear enemy effect’, during which individuals discriminate familiar neighbours from unknown strangers [26]. We analysed subject males’ aggression frequency to evaluate the dear enemy effect in our experiment.

### Procedure of experiment 2

For experiment 2, we took photographs of both sides of the eight familiar neighbours and strangers, which had never encountered these focal males, by using a camera (Sony DSC-HX200) after anaesthetization (FA100, Tanabe Pharmacy Inc., Japan). We used diluted FA100 solution, and fish colouration did not change before and after the treatment. These images were then modified by exchanging the facial areas between familiar neighbours and strangers (Fig 1B) using PowerPoint 2008 (Microsoft Corporation). The facial area contains colouration of two dark brown dots (one behind the eye and another one on the edge of gill-cover), a yellow dot above the brown dots, and light blue mesh patterns on the cheek (Fig 1A). We defined the facial colouration of *N*. *pulcher* as the area of the set of two brown dots, a yellow dot and a light blue mesh. Most fish have more photoreceptors than humans [28]. Thus, we took UV photographs of the whole body of these fish with black light, but the photos suggested that specific colouration of UV did not appear on the body or facial area of these fish (Kohda pers. obs.).

We made four digital-models of four face/body combinations for each of eight subject males: familiar neighbour’s face and body (FfFb), familiar neighbour’s face on stranger’s body (FfSb), unfamiliar stranger’s face and body (SfSb) and stranger’s face on familiar neighbour’s body (SfFb). We exchanged facial areas between a familiar neighbour fish and stranger fish that had a similar colour tone on the whole body surface. Furthermore, slight adjustment of body colour tone was performed using PowerPoint 2008 to ensure body colouration was consistent, and the margin between the visually transplanted face and body was not obvious to human observers (Fig 1B).

After an adjacent neighbour’s tank was shifted, a monitor display (FlexScanS1932, Nanao) was set on the same side of the neighbour tank, and a digital image of an empty experiment-tank with aeration under the same lighting conditions was exhibited for two days for fish to become accustomed (Fig 1C). The focal fish immediately resumed normal swimming behaviour in the same manner as when housed alongside an actual empty tank. Two days later, digital fish-models were presented to focal individuals. One of the four models was presented at random once for one minute every three days in total of four times during 10 days. The models appeared from behind a right solid square block and progressed horizontally and slowly to the centre of the tank where it paused for 5 seconds before moving behind the left solid block. This process took 15 seconds, and was repeated four times in opposite directions so that the total exposure time to the digital stimulus was one minute. During model movement, the focal fish’s behavioural responses to the model were videotaped from front side in parallel with screen (Fig 1D).

We used the behaviour ‘Still: No locomotion or movement of the pectoral fins’ for characterizing behaviour [30]. Addiotionally we define ‘watching’ behaviour as a modification of this, in which an individual assumed a stationary position while maintaining visual contact with both eyes after rapid approaching a visual stimulus (Fig 1D). This behaviour was distinct from a simple pause in swimming but with moving fins, and was not a typical threat display.

Focal fish rarely tried to attack models on display but frequently performed watching behaviour, whereas focal fish did perform aggression against real fish put in the neighbour tank. This different response is likely due to differences in movements between the digital model fish (which move steadily ahead) and real fish that also engage in reciprocal aggression with the focal fish. However, even if responses towards the digital models are different from those against real fish, the assay we used here nevertheless provides a metric for analysing fish recognition of certain aspects of visual cues in these models. We measured the time focal animals performed watching behaviour as an indicator of recognition. We predicted that duration of watching behaviour would be higher for stranger model (SfSb) than familiar neighbour model (FfFb) because fish exercised more aggression and caution to the strangers than live familiar neighbours. If *N*. *pulcher* recognize individuals based on facial features only, their response to models of familiar neighbours’ faces regardless of body (i.e. FfFb and FfSb) and responses to strangers’ faces regardless of body (i.e. SfSb and SfFb) will be similar. If they use both facial colouration and body characteristics for individual recognition, it will be predicted that the response to modified models (FfSb and SfFb) will be rather intermediate between that of unmodified models (SfSb and FfFb).

Upon focal fish noticing the digital-model that emerged from behind the black square at the first trial, they rapidly approached the model, stopped and watched the model (Fig 1D). Initial observations suggested that the distance from which the focal fish observed the model at first emergence was different between familiar-face models and unfamiliar-face models. If so, this distance will be another parameter that may indicate whether fish distinguish familiar neighbour models from unknown stranger models. Thus, we could measure the distance between models and the site at which focal fish watched the model at the initial emergence of the models in many cases (Fig 1D). We could observe the start of dash to the model after confirming the timing at which the fish noticed the emerging model in some cases. These fish started to dash to models instantly after noticing the model.

### Statistical analyses

All statistical analyses were performed with package lme4 in *R* (*R* core team). As data sets in this study were non-parametric, we used Wilcoxon signed-rank tests for dependent data and Mann Whitney *U*-tests for independent data.

### Ethics statement

We did not sacrifice study animals during our experiments. We provided food once per day and kept the fish in good aquarium conditions. Diseases were observed several times, and these diseased fish individuals were treated with medicine (Green-F, Sanei Pharmacy Inc., Japan) and used after recovery. Our experiments were conducted in compliance with the Guideline of Animal Welfare of Japan Ethological Society, and were specially approved by the Animal Care and Use Committee of Osaka City University.

## Results

### Experiment 1

The duration of aggression by males across the glass dividers was high on the first day (31.1 sec ± 9.9SD/ min, *n* = 8), but decreased steadily to a low level after 7 days (4.3 sec ± 0.58SD/ min; Wilcoxon signed-rank test, *Z* = 2.886, *p* = 0.004). An unfamiliar ‘stranger’ male presented on the 10th day to each focal individual was attacked significantly longer (20.5 sec ± 7.3SD/ min) than the familiar individual after 7 days (*Z* = 2.886, *p* = 0.004), indicating dear enemy relationships may be established within 7 days of initial interactions between the focal fish and neighbours.

### Experiment 2

**Fig 2:**
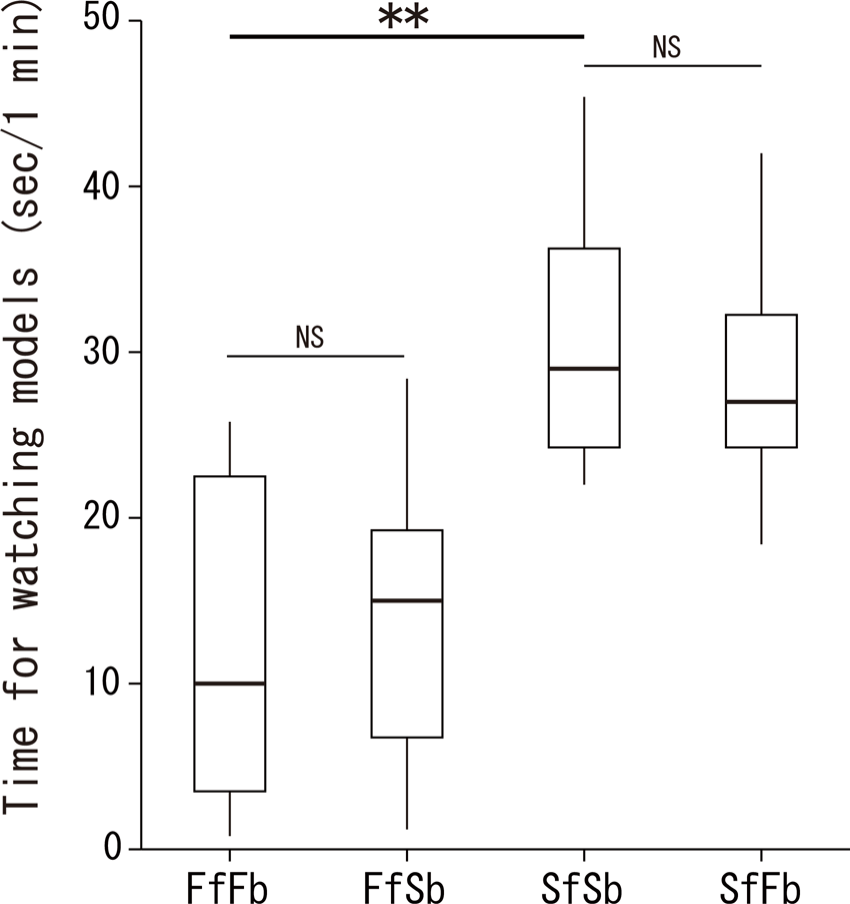
Time of watching models by *Neolamprologus pulcher* when presented four types of digital models. (FfFb): familiar neighbour, (FfSb): familiar neighbour’s face on stranger’s body, (SfSb): stranger, (SfFb): stranger’s face on familiar neighbour’s body. Median, box (showing 25% and 75%) and ranges are shown. **p < 0.01, NS *p* > 0.05. (Wilcoxon signed rank test).

Focal fish spent less time watching familiar neighbour models (FfFb) than stranger models (SfSb) (Wilcoxon signed-rank test, *Z* = 2.266, *p* < 0.02, *n* = 8, Fig 2), and they did not differentiate among models with the same face but different bodies (FfFb vs. FfSb, *Z* = 0.421, *p* = 0.73; SfSb vs. SfFb, *Z* = 0.141, *p* = 0.96, *n* = 8). In contrast to our prediction, the duration of watching modified models (FfSb & SfFb) was not intermediate between original familiar neighbour models (FfFb) and stranger models (SfSb) (Fig 2). This pattern indicates that focal fish use facial colouration as a signal for individual discrimination rather than bodily traits.

**Fig 3:**
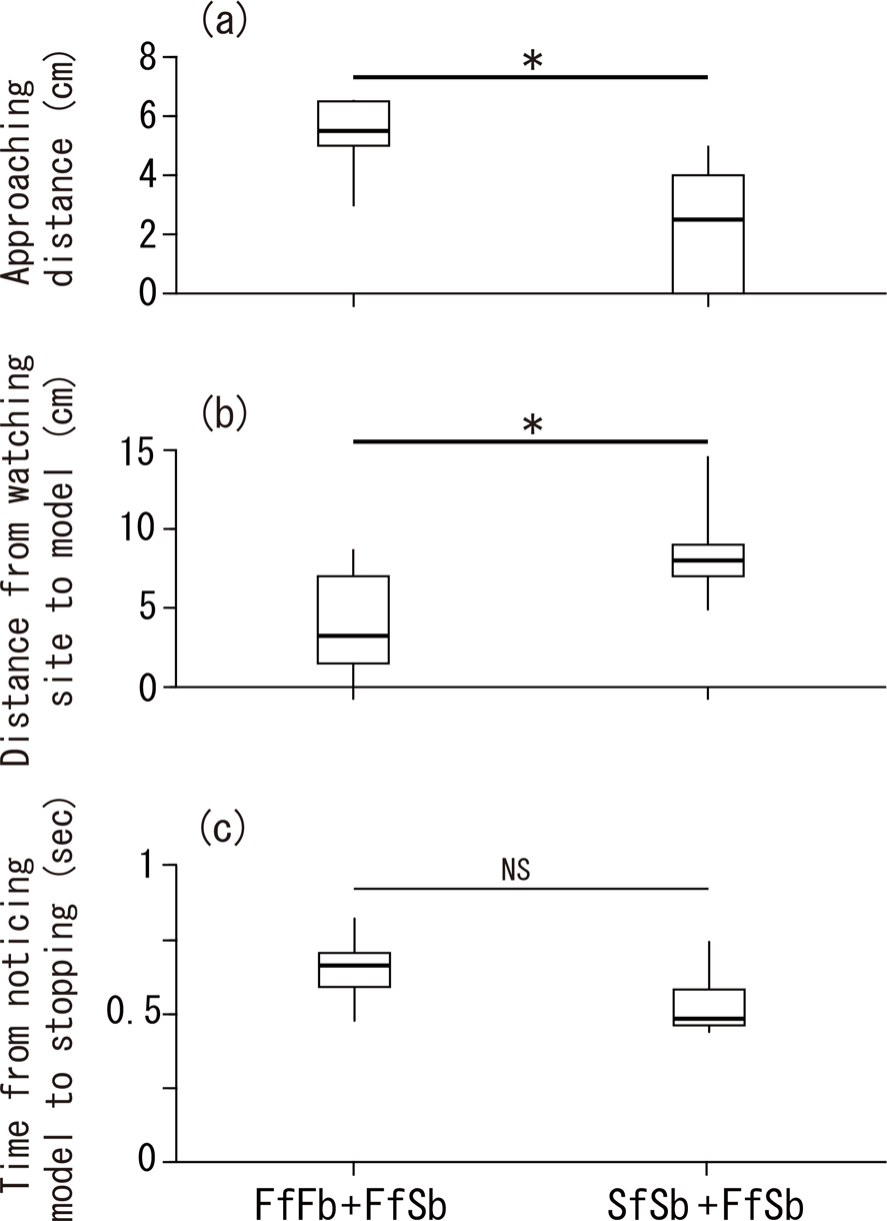
Approaching distance and time to respond to models that initially emerged from behind a right solid block after noticing the models. (a): approaching distance from site of noticing emerging models to site of watching the model [(1) shown in Fig 1D], (b): distance between the site of watching model and model on screen [(2) in Fig 1D], and (c): time from noticing the emerging model to stopping at the watching site. Median, box (showing 25% and 75%) and ranges are shown. *p < 0.05, NS *p* > 0.05. In (a) and (b), familiar face models (FfFb or FfSb) and unfamiliar face models (SfSb or SfFb) are compared. When both data are available in a fish, FfFb and SfSb are used. [Wilcoxon signed rank test in (a) and (b), and Mann Whitney *U*-test in (c)].

When the digital model emerged from the black square first time, there was no difference in the distance between the focal fish and the digital model of familiar neighbour-face models (FfFb and FfSb, 8.9 cm ± 3.1SD, *n* = 16) and stranger-face models (SfSb and SfFb, 10.7 ± 3.1, *n* = 16, Mann-Whitney *U*-test *U* = 85, *p* = 0.10). On noticing the models emerging from the square at the first time in each trial (*n* = 32, four models x 8 fish), fish often approached the model rapidly, stopped and watched the model in front of the glass wall (Fig 1D). Fish approached more and watched familiar neighbour-face models at a shorter distance than stranger-face models (Wilcoxon signed-rank test, *Z* = 2.555, *p* = 0.01, *n* = 8, 5.3 ± 3.0 in familiar face, and 2.5 ± 2.5 SD in unfamiliar face in approach distance; *Z* = 2.254, *p* = 0.024, *n* = 8, 3.8 ± 2.5 SD in familiar face and 8.8 ± 3.2 SD in unfamiliar face in distance between model and watching site, Fig 3A and 3B). This difference in distance was consistent with the difference in watching time between familiar face models and unfamiliar stranger face models, strongly suggesting that fish rapidly distinguish facial patterns upon seeing them.

In 11 of 32 cases, the time taken by fish to notice the initially emerging models and initially approaching the model could be measured from the video recordings (to a precision of 0.01 second). There was no difference in the time spent moving after noticing emerging models between familiar face models (0.64 sec ± 0.13 SD, *n* = 6) and unfamiliar face models (0.53 ± 0.12, *n* = 5; Mann Whitney *U*-test, *U* = 8.0, *p* = 0.24). In both cases the reaction times after noticing the models were very small (Fig 3C).

## Discussion

The cooperatively breeding cichlid *Neolamprologus pulcher* has been reported to distinguish familiar and unfamiliar fish, seemingly based on individually specific visual signals [24, 25, 26]. Our results using digital models are in line with these results, demonstrating that focal fish distinguish familiar neighbours from unknown strangers on digital displays (Fig 2). We expand on this result by showing that facial features are the signals these fish use to discriminate among individuals: the observed behavioural responses to models were clearly dependent on facial features, but were not affected by bodily traits (Figs 2, 3A and 3B). Our experiments are the first to exchange facial and bodily visual cues using digital manipulation, and this is the first report describing the visual signals used in individual discrimination in fish.

The sources of selection on facial patterning in *N*. *pulcher* have been discussed by Duftner et al. 2007 [31]. The authors argue that facial patterns likely do not function in mate choice (as they are sexually monomorphic), and are not the result of random processes because intra-population variation is seemingly low. Rather, they suggest that facial patterns may be mainly shaped by natural selection as signals of identity at the intra-population and intra-family levels. Here, we directly test this hypothesis and provide evidence in support of a communication role at an even more sophisticated level—at that of the individual.

In animal societies where social interactions occur frequently and repeatedly between two individuals over long time periods, individual recognition among group members will be advantageous. Traits that confer rapid and accurate identification of individuals may be favoured in both signaller and receiver [8, 9, 11, 12]. Rapid and accurate identification can be advantageous for individuals for the maintenance of social hierarchies to avoid the costs of repeated escalation of contests and disputes. Our results show that *N*. *pulcher* can distinguish individuals extremely rapidly and accurately. In *N*. *pulcher*, individual social groups are located amongst larger colonies, and border disputes and interactions with conspecific rivals are common [23]. Under these conditions, *N*. *pulcher* frequently encounter familiar and unfamiliar social partners and must perform suitable social interactions, for example, tolerance or aggression in context of dear enemy relation in territories and/or appeasement displays toward dominants.

On noticing emerging models, *N*. *pulcher* rapidly approached the models, and stopped in front of the models at a shorter distance for familiar neighbour-face models than stranger-face models. This difference also indicates that fish distinguish fish-models depending on facial colour patterns, and suggests that fish might distinguish familiar neighbours and strangers before stopping at the watching site. Importantly, in both familiar-face model and unfamiliar stranger-face model, fish approached and stopped at the watching site in less than 0.6 sec after noticing models, indicating that this fish distinguishes two types of model in short time (0.4 sec). This indicates that *N*. *pulcher* can distinguish familiar neighbours and unfamiliar fish accurately and extremely rapidly. Our results suggest this rapid visual assessment can be achieved as effectively as in other documented examples of recognition in vertebrates. However, the timing of recognition needs further explicit study to investigate how rapidly fish can discriminate familiar and unfamiliar fish in detail.

Fish may use olfactory, chemical and visual cues for individual identification [21], but in aquatic habitats of high transparency, such as coral reefs or shallow shores of tropical lakes, visual cues are expected to be most salient. Thus, it is plausible that, in these waters, many highly social fish will initially discriminate group members visually, and these fish might then develop visual signals for individual recognition [8, 24, 25]. Interestingly, in Lake Tanganyika, cooperative breeding cichlids living in large social groups in shallow waters tend to have colouration in the facial area (including the area around the eye and gill cover) but not in other body parts. Of 11 cooperative breeding cichlids of the genus *Neolamprologus* [32], seven species have clear species-specific facial colouration, which are also individual-specific (e.g. *N*. *brichardi*, *N*. *obsculus*, *N*. *savoryi*, *N*. *splendens*, for facial colouration, see [33]). In contrast, in all seven species of monogamous *Neolamprologus* cichlids without helpers, no species have such prominent facial colour patterns (i.e. *Neolamprologus caudopunctatus*, *N*. *leleupi*, *N*. *leloupi*, *N*. *sexfasciatus*, *N*. *tetracanthus*, *N*. *toae*, *N*. (*Variabilichromis*) *moorii*) [33]. Conspicuous colouration may be costly, e.g. risk of attracting predators [5, 6], and may therefore be faint or absent if the benefits do not outweigh the costs [34]. Permanently territorial fish frequently encounter multiple territorial neighbours, and may thus be highly social [13]. Of such territorial damselfish, two sympatric species, *Pomacentrus amboinensis* and *P*. *moluccensis*, have visible face colouration in the ultraviolet spectrum, and use it for species recognition and potentially for individual recognition [34, 35]. Similar patterns of facial colouration are likely found in other permanently territorial damselfishes (e.g. *Paraglyphidodon polyacanthus*, *Stegastes nigricans*, *S*. *apicalis* in [36]), whereas such facial colouration is likely not to appear in gregarious damselfish [36]. Together with this information and our results, we expect that the facial colour patterns of social fish, at least these cooperatively breeding cichlids and permanently territorial damselfish, will be used as signals for individual recognition in societies where individuals will need to discriminate several conspecific individuals.

We propose three hypotheses to explain why individually specific colouration tends to develop in the facial regions; (1) When fish encounter each other, they will often approach in a ‘head to head’ position, where signals on the frontal parts of the fish body will be effective. The colour pattern used as signal, however, does not appear in the head area between the eyes in either *Neolamprologus* spp. or the territorial damselfishes including *P*. *amboinensis* and *P*. *moluccensis*, but rather appear in the facial side area near the eye. This suggests that the frontal part of the body is not inevitably important for the location of individual signals. (2) The critical social signal should be on the major parts of the body, but not on peripheral parts that could easily be damaged or lost in conflict, such as on the fins. However, in this case, individual-specific cue would not necessarily be restricted to the facial area but could also occur on the central part of their trunk. Indeed, species-specific sexual colourations used primarily as indicators of male quality rather than for individual recognition, are located on the body in some model fish species (bright red colour on the belly of three-spine stickleback [37]; orange spot on the flanks of the guppy [38]; white patch on the frontal trunk in blue-head wrasse [39]). However, the visual signals for individual recognition in fish appear to be restricted to the facial area around the eye or within the area from the snout to the gill cover ([33, 34, 35, 36] and present study). Thus, this hypothesis cannot exclusively explain the facial colour patterns used as individual cues. (3) If fish initially attend to the eyes of other individual fish rather than other parts of the body, the social signal around eye will be advantageous for rapid signalling for both signal senders and receivers [8]. Social mammals, e.g. chimpanzee, are likely initially watching the eyes of other individuals, and identify them using facial patterns (e.g. [14]). Similar studies of eye tracking, however, have not been done in fish. In addressing these hypotheses, we find that the individual cues in cichlids and damselfishes are located near the eye or in facial area, rather than on the head between the eyes (rejecting hypothesis 1), and do not occur other parts of the body (rejecting hypothesis 2), suggesting that hypothesis 3 could be most plausible. However, this needs to be verified in future studies.

The accuracy and speed with which *N*. *pulcher* can immediately discriminate slight differences in facial colour patterns between models suggests a cognitive capacity for social recognition comparable to facial recognition by monkeys (e.g. [40]], apes [14] or humans. Primates and sheep have specific neural mechanisms for visual recognition of faces (e.g. [40, 41, 42]), and the accuracy and the rapidity of facial recognition of *N*. *pulcher* suggest that they might have similar neural capacities to discriminate facial features. Recently, various kinds of enhanced social cognitive abilities, previously reported only from mammals, have been documented in fishes [13, 19, 20, 21], and the present study adds a novel example of social cognition in fish.
